# The effect of *Melissa officinalis* syrup on patients with mild to moderate psoriasis: a randomized, double-blind placebo-controlled clinical trial

**DOI:** 10.1186/s13104-021-05667-9

**Published:** 2021-06-30

**Authors:** Alireza Yargholi, Leila Shirbeigi, Roja Rahimi, Parvin Mansouri, Mohammad Hossein Ayati

**Affiliations:** 1 Department of Persian Medicine, School of Persian Medicine, Tehran University of Medical Sciences, Tehran, Iran; 2 Department of Traditional Pharmacy, School of Persian Medicine, Tehran University of Medical Sciences, Tehran, Iran; 3 Department of Dermatology, Faculty of Medicine, Tehran University of Medical Sciences, Tehran, Iran; 4 Department of Traditional Medicine, School of Persian Medicine, Tehran University of Medical Sciences, P. O. Box: 1416643139, Tehran, Iran

**Keywords:** Psoriasis, Quality of life, Pruritus, *Melissa officinalis* syrup

## Abstract

**Objective:**

Psoriasis is an immune-mediated inflammatory skin disease. It can involve any body skin area, particularly the scalp, lower back, elbows, and knees. There are several topical and systemic therapies for the treatment. Nowadays, herbal medicines are popular treatments for dermatologic conditions. This two-arm parallel, randomized placebo-controlled clinical trial was conducted to examine the hypothesis of the efficacy of *Melissa officinalis* syrup on patients with mild-to-moderate Plaque psoriasis.

**Result:**

Among 100 patients, 95 participants completed the trial and five of them withdrew. The mean pruritus intensity and PASI scores decreased significantly in the intervention group compared to the placebo group (P < 0.001). The DLQI score in the intervention group increased post-treatment compared to pre-treatment (P = 0.029); however, there was no significant difference between the intervention and control group at the end of the study (0.065).

*Trial registration*: The trial was registered in the Iranian registry of clinical trials on November 9th, 2019 (https://www.irct.ir/trial/43434; registration number: IRCT20191104045326N1).

**Supplementary Information:**

The online version contains supplementary material available at 10.1186/s13104-021-05667-9.

## Introduction

Psoriasis is defined as an immune-mediated inflammatory disease [1, 2]. Based on the World Health Organization reports, it is estimated that at least 100 million people have psoriasis worldwide [3]. Skin manifestations are more prominent symptom of psoriasis. It can affect any skin area, particularly the scalp, such as topical, systemic, and biologic therapies for psoriasis treatment [4–6]. Nowadays, many patients intend to use complementary and alternative medicine (CAM) due to low efficacy and side effects of conventional therapies [7]. It is estimated that 43–69% of patients with psoriasis use different modalities of CAM [8, 9], which herbal medicines are the most popular [10].

*Melissa officinalis* is one of the herbs that are theoretically effective and safe. It has traditionally been applied in skin disorders such as eczema and skin inflammation [11]. *Melissa officinalis* syrup is a mixture that contains four ingredients of Damask rose (*Rosa damascena*), Fennel (*Foeniculum vulgare*), Lemon balm (*Melissa Officinalis*), and honey. This syrup is formulated based on the Traditional Persian Medicine (TPM) texts.

While it is supposed that *Melissa officinalis* syrup is a compelling mixture for the improvement of psoriasis in theory, but the evidenced-based efficacy of it was unclear. Therefore, this randomized placebo-controlled clinical trial was designed to investigate the effectiveness of this formula on patients with psoriasis.

## Main text

### Methods

The present study is a 12-week, randomized, double-blind, parallel control trial carried out in the Skin and Stem Cell Research Center affiliated to Tehran University of Medical Sciences between November 2019 and May 2020. The trial was registered at the Iranian registry of clinical trials on November 9th, 2019 (https://www.irct.ir/trial/43434; registration number: IRCT20191104045326N1). A written consent form was taken from eligible patients before recruitment in the trial.

### Participants

We recruited patients with mild to moderate psoriasis, aged over 18 years old. Participants with the following conditions were included: a Psoriasis Area and Severity Index (PASI) of < 30%, and a body surface area (BSA) of < 10%, clinically diagnosed Plaque psoriasis, controlled psoriasis during last two months, not using Psoralen and phototherapy during the past 4 weeks, not participating in other studies during the previous month and during the study. Patients were excluded if they (1) had different types of psoriasis such as psoriatic arthritis, pustular psoriasis, erythrodermic psoriasis, etc. (2) used corticosteroids and immunosuppressive drugs and systemic therapies during the last 4 weeks, (3) using some medications that may intensify psoriasis including beta-blockers, Calcium channel blockers, non-steroidal anti-inflammatory drugs, lithium, etc. (4) patients with cardiovascular and cancer diseases, (5) allergy to any components of syrup, (6) pregnancy and lactation, (7) Acute progressive psoriasis and erythroderma tendency.

### Sample size estimation

Based on previous studies and considering the following formula as the sample size equation for comparing two populations, p1 = 15%, p0 = 40%, α = 0.05, β = 20%, the sample size was estimated at 45 individuals. Finally, considering a 10% loss to follow-up, 50 individuals were recruited in each group.

$$N = \;\frac{{2\left( {Z_{{1 - \alpha /2}} + Z_{{1 - \beta }} } \right)^{2} \times p\left( {1 - p} \right)}}{{\left( {po - p1} \right)^{2} }}\;\;\;\;\;\;\;~P = \frac{{po + p1}}{2}.$$

### Randomization and intervention

Participants were randomly assigned to the intervention or control group using the random number sequence function in Excel and a 1:1 allocation ratio. Patients in the intervention group consumed two tablespoons (each tablespoon = 10 ml) of *Melissa officinalis* syrup three times per day (two tablespoons before each meal of breakfast/ lunch/ dinner) and patients in the control group received the same amount of placebo.

### Drug preparation

*Melissa officinalis* syrup contained 3 g of Lemon balm, 8 g of Damask rose, and 1 g of Fennel. Firstly, the decoction of these herbs was prepared. The herbs and 450 ml water were added to a pot and heated to boil gently. It boiled until the volume is reduced to 250 ml. While boiling, some honey was added to sweeten it. The placebo syrup contained sugar, caramel coloring additive, and 1% rose water. The pharmacy department of traditional medicine college prepared both *Melissa officinalis* syrup and placebo. There is no detectable difference between *Melissa officinalis* syrup and placebo syrup in color, odor, viscosity, and containers. Patients were monitored by a practitioner and they were able to ask their questions anywhere and anytime by telephone or face-to-face in the clinic. The duration of intervention was 12 weeks and patients were checked during this period for any potential side effects.

### Total phenolic and total flavonoid assay

The total phenolic content was determined by using the Folin-Ciocalteu assay and was expressed as mg Gallic acid Equivalents (GAE). Total flavonoid content was measured by the aluminium chloride colorimetric assay and was expressed as mg quercetin equivalents (QE) [12]. Total phenolic and flavonoid contents of each ml of syrup were estimated to be equivalent to 185.22 ± 0.71 mg gallic acid and 103.64 ± 1.32 mg quercetin, respectively.

### Acute toxicity study

Wistar rats were used for the assessment of acute toxicity of syrup [13]. Considering the results, the syrup shows no acute toxicity up to the dose of 5 ml/kg. This dose is corresponding to 0.81 ml/kg in human [14].

### Outcomes

The tools used for data collection were a socio-demographic questionnaire, Psoriasis Area and Severity Index (PASI), Life Quality Index (DLQI) questionnaire, and Visual Analogue Scale (VAS). Photographs of each subject was taken at the baseline, during, and end of study. PASI combines the severity of lesions and the body surface area involvement into one score. It has a range score from 0 (no disease) to 72 (maximum severity of the disease) [15]. DLQI questionnaire is designed for adult patients with skin diseases and is a 4-point Likert scale questionnaire that each question gets a score from zero (not at all) to three (very much). The total DLQI score range from 0 to 30, that higher scores represent higher impairment of quality of life [16]. A VAS scale measured the pruritus intensity. Patients draw a line on 0–10 cm VAS scale to determine their itching level at the beginning and end of the study. It is a reliable and valid instrument that is most widely used in epidemiologic and clinical trials. It has a range score from 0 to 10, of which 0 expresses no pruritus and 10 depicts intense pruritus [17].

### Statistical analyses

We used SPSS version 19 (SPSS Inc., Chicago, IL, USA) for windows to perform data analysis. P values under 0.05 were regarded as significant. Categorical data were expressed in percentage (%), while continuous data were expressed in mean ± SD in evaluating patients’ demographic details. Chi-square or Fisher exact test was used for the categorical data and Independent Samples t-Test was used for continuous data to show the differences. For comparison of PASI, DLQI, and VAS parameters between intervention and placebo groups, an independent t-test was applied at the first and end of the study. A paired sample t-test was also used to compare the mean of variables at pre and post-treatment in each group.

## Results

**Fig. 1:**
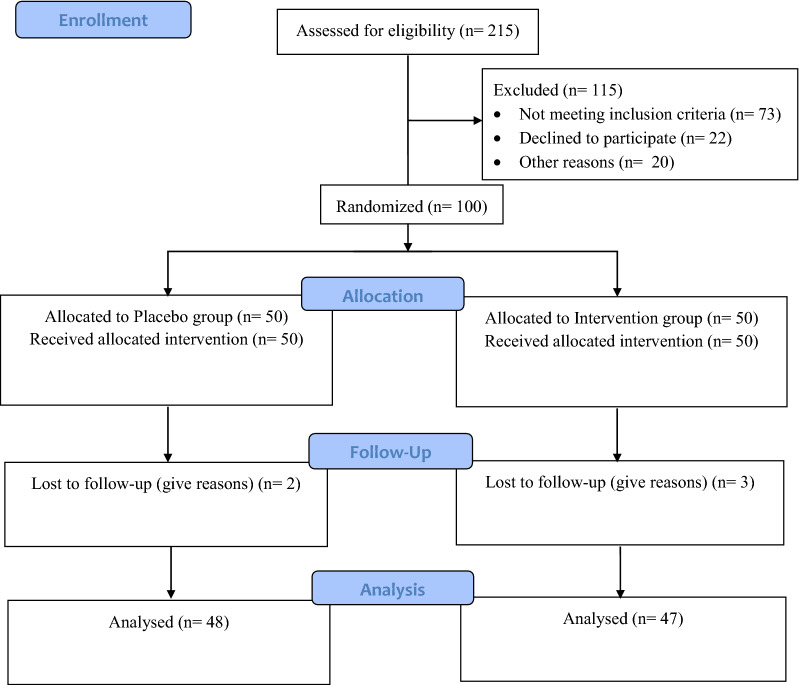
Flow diagram of the study

Among 215 patients examined for eligibility, one hundred participants met the inclusion criteria that were randomized into intervention (n = 50) or placebo (n = 50) groups. At the end of follow-up, five patients (3 in the intervention group and 2 in the placebo group) withdrew and 95 individuals completed the trial (Fig. 1). There was no significant difference between intervention and placebo groups in terms of baseline variables (Table 1).

**Table 1:** Demographics characteristics of participants at the baseline of study

Variables	Intervention group (n = 50)	Control group (n = 50)	p-value
Age (year)	39.52 ± 14.57	39.29 ± 12.03	0.933^a^
Height (cm)	166.97 ± 11.54	169.27 ± 12.54	0.361
Weight (Kg)	72.50 ± 23.14	72.27 ± 16.70	0.956
BMI (Kg/m^2^)	25.81 ± 7.29	25.01 ± 4.51	0.526
Gender			0.217
Male	18 (37.5%)	24 (50.0%)	
Female	30 (62.5%)	24 (50.0%)	
Smoking			0.136
Yes	4 (8.3%)	9 (18.8%)	
No	44 (91.7)	39 (81.2%)	
Alcohol			0.242
Yes	3 (6.2%)	0 (0.0%)	
No	45 (93.8%)	48 (100%)	
History of diseases			0.066
Yes	17(35.4%)	9 (18.8%)	
No	31 (64.6%)	39 (81.2%)	
Comorbidity			0.789
Yes	9 (18.8%)	8 (16.7%)	
No	39 (81.2%)	40 (83.3%)	
History of medication			1.00
Yes	44 (91.7%)	45 (93.8%)	
No	4 (8.3%)	3 (6.2%)	
Duration of disease			0.440
< 1 years	6 (12.5%)	9 (18.7%)	
1–5 years	13 (27.1%)	13 (27.2%)	
5–15 years	16 (33.3%)	11 (22.9%)	
> 15 years	13 (27.1%)	15 (31.2%)	
Numbers of area			0.146
1	8 (16.6%)	16 (33.3%)	
2	9 (18.7%)	13 (27.1%)	
3	12 (25.0%)	8 (16.7%)	
4	8 (16.6%)	5 (10.4%)	
≥ 5	11 (22.1%)	6 (12.5%)	

Values are presented as mean ± SD for quantitative variables and n (%) for qualitative variables

^a^P-value is calculated by the independent t-test

^b^P-value is calculated by and Chi-square test

Results indicated that no notable differences in any scales between the two groups were found at the study’s baseline (P > 0.05). The mean pruritus intensity score decreased significantly from 6.34 ± 2.68 at the baseline of the study to 3.45 ± 2.18 at the end of follow-up (P < 0.001), which was considerably lower than the placebo group (P < 0.001). While pruritus intensity score did not change significantly in the placebo group (P = 0.886). Moreover, patients in the intervention group had significantly lower PASI score than patients in the placebo group at the end of the study (1.65 ± 2.17 versus 3.36 ± 2.38; P < 0.001). Lesion improvement in the arm area of the patients are demonstrated in Additional file 1: Figure S1. The DLQI score was 67.63 ± 22.97 in the *Melissa officinalis* group and 64.43 ± 23.66 for the placebo group (P = 0.503). After a 12-week intervention, the scores were changed to 74.37 ± 23.89 and 65.29 ± 23.74, respectively (Table 2).

**Table 2:** Results of itch score, PASI score, and DLQI questionnaire between intervention and control groups

Variables	Intervention group (n = 47)	Placebo group (n = 48)	P-value^a^
	Mean ± SD	Mean ± SD	
Itch			
Pre-treatment	6.34 ± 2.68	5.45 ± 2.55	0.104
Post-treatment	3.45 ± 2.18	5.56 ± 2.81	< 0.001
p-value^b^	< 0.001	0.886	
PASI			
Pre-treatment	3.26 ± 3.58	2.95 ± 2.16	0.603
Post-treatment	1.65 ± 2.17	3.36 ± 2.38	< 0.001
p-value	< 0.001	0.002	
DLQI			
Pre-treatment	67.63 ± 22.97	64.43 ± 23.66	0.503
Post-treatment	74.37 ± 23.89	65.29 ± 23.74	0.065
p-value	0.029	0.519	

*DLQI* dermatology life quality index, *PASI* psoriasis area and severity index, *SD* standard deviation

^a^P-value is calculated by the independent t-test

^b^P-value is calculated by the Paired t-test

## Discussion

This trial indicated that *Melissa officinalis* syrup could significantly improve the treatment of psoriasis through reducing scores of PASI and itch in patients with mild to moderate severity.

The first-line of psoriasis treatment is usually topical treatments such as vitamin D analogues and corticosteroids [18]. Furthermore, some topical herbal medicines such as aloe vera, cayenne, chamomile, turmeric, and flaxseed oil can likely have beneficial effects on psoriasis management [19]. besides, TPM recommends several topical remedies, including oil of wheat germ, flaxseed, black seed, and violet [20].

In addition to topical use, some herbal medicines are used as oral administration. A review of clinical trials was conducted by May and colleagues to assess herbal medicines’ systemic use in psoriasis. They identified 12 clinical trials, that eight of them were multi-ingredient herbal formulations [21]. Moreover, a meta-analysis by Zhang et al. indicated that oral Chinese herbal medicines could be useful in the treatment of psoriasis [22]. TPM recommend *Melissa officinalis* syrup, which contain three herbs of Lemon balm, Fennel, and Damask rose.

Lemon balm (*Melissa officinalis*) is one of the well-known herbal medicines used in traditional medicine all over the world [23]. Dimitris et al. carried out an experimental study to investigate the effects of *Melissa officinalis* on psoriasis in mice. They concluded that dichloromethane extract and decoction of *Melissa officinalis* could prevent epidermal hyperplasia and scaling, while methanol extract did not have a significant effect. A valuable finding was that the decoction was more effective as compared to dichloromethane extract that approved the traditional use of lemon balm decoction as an anti-psoriatic [24].

Damask rose (*Rosa damascena*) is an herbal medicine used in TPM for different conditions, including abdominal pain, digestive disorders, wound healing, and skin health [25]. Shabikin et al. found that topical rose hip seed oil combined with oral fat-soluble vitamins could be useful in different inflammatory dermatitis such as eczema, neurodermatitis, and cheilitis [26].

Fennel (*Foeniculum vulgare*) is a well-known medicinal plant and have had a widespread application traditionally in different conditions such as respiratory and gastrointestinal problems, arthritis, and mouth ulcer in many parts of the world. An experimental study demonstrated that Fennel has anti-inflammatory and antimicrobial effects and enhance synthesis of collagen, which might be useful in wound healing. Furthermore, Fennel is a rich source of phytoestrogens, which are able to promote collagen and water content and protect the skin from oxidative stress [27].

How this formulation could improve psoriasis symptoms is not entirely known. Psoriasis is a disease with inflammation origin [28]. A number of previous studies indicated anti-oxidant, anti-inflammatory, and immunomodulatory activities of *Melissa officinalis*, damask rose, and fennel [25, 29, 30]. These herbs are a rich source of phenolic compounds such as flavonoids, cumarin, tannin, caffeic acid, caffeic acid, and rosmarinic acid [31–33]. A study confirmed the therapeutic effects of rosmarinic acid on atopic dermatitis [34]. Also, another study demonstrated that decoction of *Melissa officinalis* has a lot of caffeic acids and rosmarinic acid, whose synergistic effects resulted in significant improvement of psoriasis [24].

In summary, treatment with *Melissa officinalis* syrup demonstrated a significant reduction in pruritus and PASI scores and improved quality of life. The findings of this study may be useful for patients with mild-to-moderate chronic plaque psoriasis.

### Limitations

This study has some inevitable limitations including (1) due to lack of post-trial follow-up, long term effects of *Melissa officinalis* syrup is not ascertained; (2) using questionnaire may lead to bias; (3) participants were only patients with mild to moderate plaque-type psoriasis and patients with severe psoriasis and other types of psoriasis were not included; (4) biochemical tests were not considered because of resources limitations and they are proposed to be measured in future trials.

## Supplementary Information

**Additional file 1: Figure S1.** Improvement in psoriasis plaque on the arm over 12 weeks treatment: A) week 0, B) week 4, C) week 8, D) week 12.

## Data Availability

The study protocol and data collection forms are available from the corresponding author (MHA) upon reasonable request. The datasets generated and analyzed during the current study are not available to researchers outside of the co-investigators due to data protection laws.

## Abbreviations

BSA: Body surface area
CAM: Complementary and alternative medicine
DLQI: Dermatology Life Quality Index
PASI: Psoriasis Area and Severity Index
VAS: Visual Analogue Scale
