# Supplementary material for: The effect of Melissa officinalis syrup on patients with mild to moderate psoriasis: a randomized, double-blind placebo-controlled clinical trial
Source: BMC Res Notes. 2021 Jun 30;14:253. doi: 10.1186/s13104-021-05667-9 (PMC8246655; doi:10.1186/s13104-021-05667-9)
Supplement: Supplementary file 1 — Additional file 1: Figure S1. Improvement in psoriasis plaque on the arm over 12 weeks treatment: A) week 0, B) week 4, C) week 8, D) week 12. [file 13104_2021_5667_MOESM1_ESM.docx]

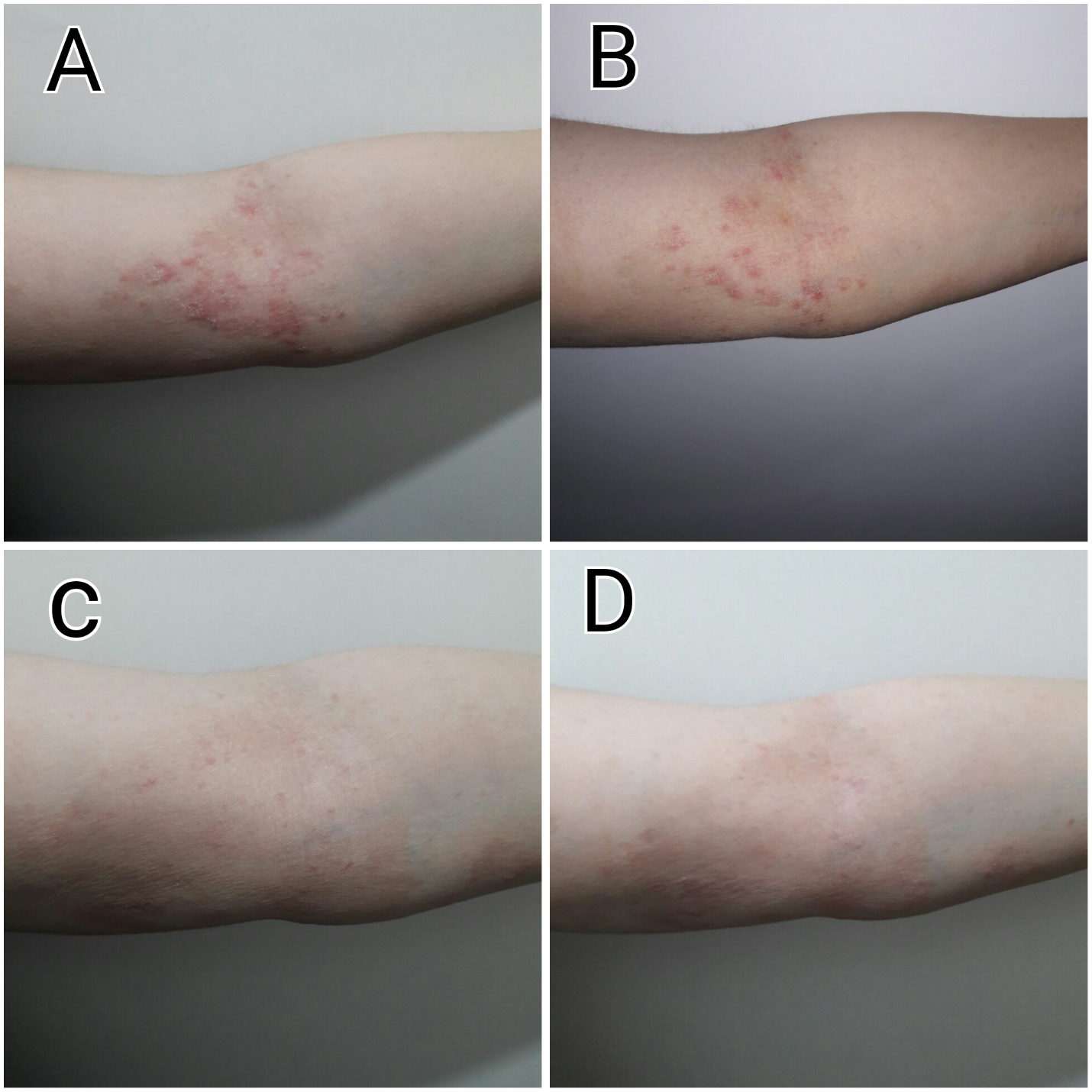


**Figure S1.** Improvement in psoriasis plaque on the arm over 12 weeks treatment: A) week 0, B) week 4, C) week 8, D) week 12
